# Between-Day Reliability of Commonly Used IMU Features during a Fatiguing Run and the Effect of Speed

**DOI:** 10.3390/s22114129

**Published:** 2022-05-29

**Authors:** Hannah L. Dimmick, Cody R. van Rassel, Martin J. MacInnis, Reed Ferber

**Affiliations:** 1 Faculty of Kinesiology, University of Calgary, Calgary, AB T2N 1N4, Canada; crvanras@ucalgary.ca (C.R.v.R.); martin.macinnis@ucalgary.ca (M.J.M.); rferber@ucalgary.ca (R.F.); 2 Faculty of Nursing, University of Calgary, Calgary, AB T2N 1N4, Canada; 3 Cumming School of Medicine, University of Calgary, Calgary, AB T2N 1N4, Canada

**Keywords:** inertial measurement unit, running fatigue, maximal lactate steady state, running biomechanics

## Abstract

The purpose of this study was to determine if fatigue-related changes in biomechanics derived from an inertial measurement unit (IMU) placed at the center of mass (CoM) are reliable day-to-day. Sixteen runners performed two runs at maximal lactate steady state (MLSS) on a treadmill, one run 5% above MLSS speed, and one run 5% below MLSS speed while wearing a CoM-mounted IMU. Trials were performed to volitional exhaustion or a specified termination time. IMU features were derived from each axis and the resultant. Feature means were calculated for each subject during non-fatigued and fatigued states. Comparisons were performed between the two trials at MLSS and between all four trials. The only significant fatigue state × trial interaction was the 25th percentile of the results when comparing all trials. There were no main effects for trial for either comparison method. There were main effects for fatigue state for most features in both comparison methods. Reliability, measured by an intraclass coefficient (ICC), was good-to-excellent for most features. These results suggest that fatigue-related changes in biomechanics derived from a CoM-mounted IMU are reliable day-to-day when participants ran at or around MLSS and are not significantly affected by slight deviations in speed.

## 1. Introduction

Inertial measurement units (IMUs) have gained popularity in recent years due to their ability to measure gait patterns in an accessible, inexpensive, and portable manner. IMUs have been used in clinical contexts [1,2], team sports [3,4], activity recognition [5,6], and to investigate various aspects of walking and running gait [7,8,9,10]. Moreover, a growing number of studies have employed research-grade and commercial IMUs to detect fatigue-related changes in running, using various experimental setups and sensor locations [11,12,13].

Although running is a popular modality of physical activity [14], the injury rate is extremely high [15,16]. Most running-related injuries (RRIs) are due to overuse rather than acute incidents [17,18], and the risk of overuse injury may be heightened in a fatigued state, as the resultant “atypical” mechanics lead to atypical stresses on the musculoskeletal system [19,20,21]. Bones, tendons, ligaments, cartilage, and muscles may have a lower tensile limit for the direction and magnitude of the altered mechanics, causing an accumulation of microdamage “beyond the capabilities of the specific structure” that can lead to injury [22,23]. Thus, fatigue-related alterations in biomechanics have been suggested as a potential factor in the development of RRIs [19,24,25].

IMUs placed at the center of mass (CoM) have proven particularly useful for gaining insights into fatigue-related changes in running, due to their unobtrusive positioning and ability to capture deviations related to the whole body [12]. Using a CoM-mounted IMU, a variety of statistical, regularity, dynamic, and symmetry features have demonstrated sensitivity to fatigue-related changes, both in basic statistical comparisons [12,26,27,28] and as inputs into machine learning algorithms [29,30,31]. However, most studies only perform single-day data collections, and thus it has not been determined whether these features (with the exception of root mean square (RMS) [32,33,34]) possess good day-to-day reliability during either non-fatigue or fatigue conditions.

In order to elucidate the mechanism of fatigue-related injury, it is important to first determine whether running biomechanics are consistent day-to-day, to determine if internal and external circumstances (e.g., stress, sleep, diet, time of day, and running speed) influence this dynamic. For example, Kawabata et al. [33] demonstrated that changes in speed (~±15%) had a significant effect on the RMS of the acceleration signal during non-fatigue conditions, and McGregor et al. [34] similarly demonstrated significant correlations between speed and acceleration RMS/RMS ratio (RMSR) during an incremental test.

If running gait biomechanics during fatigue are consistent day-to-day, it would support the suggestion that fatigue-related changes to gait patterns are potentially related to injury, as tissues would be regularly subjected to the same atypical forces [20]. However, if fatigued biomechanics are variable, the value of using single-trial studies to analyze fatigue-related alterations may not be particularly high and deriving meaning from these experimental designs about possible mechanisms of injury may not be possible. To our knowledge, no prior study has sought to determine the day-to-day reliability of IMU-based running biomechanics during a fatigued state. Further, few studies have effectively considered the relative running intensity when evaluating changes in running biomechanics from fatigue, particularly with respect to the maximal lactate steady state (MLSS). The MLSS threshold demarcates the heavy from the severe exercise intensity domains and represents the boundary between sustainable and unsustainable intensities of exercise [35,36,37,38]. To set exercise intensity, most prior research designs have used time trial speed [11,12,24], previous race performance [30,39], or a percentage of the maximal speed obtained during an incremental treadmill test [9], which can produce heterogeneous responses in metabolic strain [40,41]. We contend that in order to better discern the effect of fatigue on biomechanical alterations in IMU data, the employed exercise stimulus should be prescribed relative to the physiological capabilities of the individual.

Therefore, the primary purpose of this study is to determine the reliability of commonly derived IMU features used for the detection of biomechanical alterations in treadmill running fatigue. We hypothesize that most features will demonstrate good-to-excellent reliability during both non-fatigue and fatigue conditions. The secondary purpose of this study is to determine if adjustments in running speed around the MLSS boundary affect measures of reliability, and we hypothesize that reliability decreases as running speed increases and/or decreases.

## 2. Materials and Methods

### 2.1. General

Sixteen recreationally and competitively trained runners (7 female, 9 male, age = 30.1 ± 4.2 yrs, height = 174.3 ± 9.1 cm, weight = 70.5 ± 10.5 kg) provided informed consent to participate in this study, which was approved by the Ethics Board at the University of Calgary (REB20-0111). Participants were included if they were between the ages of 18 and 45 and had a recent 10 km performance of ≤50 min or ≤55 min for men and women, respectively. All participants were familiar with treadmill running, were free of medical conditions and injuries that could interfere with metabolic and cardiorespiratory exercise responses, and completed the Physical Activity Readiness Questionnaire (PAR-Q+) prior to exercise to ensure there were no identifiable contraindications to exercise.

Participants visited the lab five or six times. For all visits, participants were instructed to refrain from smoking, eating, and consuming caffeine at least 2 h prior to testing and to refrain from strenuous exercise at least 6 h prior. Participants used their own running shoes but were required to use the same shoes for each visit. Participants were weighed and measured at the beginning of each visit and familiarized with the Borg rating of perceived exertion (RPE, 6–20) scale [42]. The RPE scale has previously been used in running-related fatigue literature to detect subjective and holistic changes in an individual’s capacity to continue a task [11,25,29].

#### 2.1.1. Visit 1: Step-Ramp-Step Test

On the first visit, a modified “Step-Ramp-Step” (SRS) exercise test was performed [41]. A metabolic cart (Quark, CPET, Cosmed, Rome, Italy) was used to measure ventilatory and gas exchange variables. Participants were fitted with an Oro-Nasal 7450 V2 mask connected to a two-way non-rebreathing valve (Hans-Rudolph Inc., Kansas City, MO, USA), and a plastic hose with expired air was directed into a 7 L mixing chamber, from which ventilatory parameters were measured or derived and reported as 10 s averages. Prior to the testing session, gas analyzers were calibrated using a gas mixture of known composition (5% CO_2_, 16% O_2,_ and N_2_ balance), and turbine flow meters were calibrated using a 3 L syringe, according to manufacturer guidelines. Heart rate was measured by a chest strap placed just below the sternum (H10; Polar Electro Inc., Bethpage, NY, USA). Blood lactate concentration ([BLa]) measurements were made using a capillary blood sample from the fingertip to determine resting and peak blood lactate responses to incremental exercise. [BLa] was analyzed using the Biosen C-Line lactate analyzer (EKF Diagnostics, Cardiff, Wales) for the first 8 runners. Due to supply chain issues mid-way through the course of the study, [BLa] analyses were transitioned to the Lactate Plus (Nova Biomedical, Waltham, MA, USA) system for the remaining eight runners. Data from the SRS test was used to estimate the speed associated with maximal lactate steady state (MLSS) for experimental trials.

#### 2.1.2. Visits 2–5: Experimental Trials

Prior to the start of each experimental trial, participants were outfitted with an IMU (Blue Trident, Vicon, Oxford, UK; tri-axial accelerometer sampling rate 1125 Hz, range ±16 g) positioned between the posterior superior iliac spines with the top border of the sensor positioned on a line coincident with the inferior aspect of the iliac crest. The Blue Trident IMU is a research-grade device capable of collecting data at high frequencies and providing high-quality data. The X, Y, and Z axes were oriented in the vertical (VT; + superior), medial–lateral (ML; + to the left), and anterior–posterior (AP; + to the posterior) directions, respectively. Incline of the treadmill was set at 1% to best match the energetic cost of outdoor running [43].

Each experimental trial began with the participant performing a 5 min warmup at 1.92 m/s before increasing to the target speed. The trial was terminated when the participant reached volitional exhaustion, or at 45 min, whichever occurred first. All runners performed the initial constant-speed test at the running speed estimated to be MLSS by the SRS protocol. Follow-up visits were conducted at either 5% faster or 5% slower treadmill speeds until running tests were conducted at MLSS speed, 5% above MLSS speed, and 5% below MLSS speed. The final constant-speed treadmill running visit was a repeat trial at the running speed associated with MLSS. Each visit was separated by at least 48 h, and participants were not informed of the speed until after all experimental trials were completed. The MLSS for each runner was identified as the highest treadmill speed where at least 30 min of exercise was performed and the difference between [BLa] at 10 and 30 min was < 1 mmol·L^−1^ [35], using [BLa] measurements taken from a capillary blood sample from the fingertip. Participants provided RPE measures every 5 min and at trial termination, and verbal encouragement was provided throughout.

### 2.2. Data Processing

Overall, four experimental trials at three target speeds were included for final analyses—two trials at the estimated speed at MLSS (MLSS1/MLSS2), one at 5% faster than estimated speed at MLSS (F), and one at 5% slower than estimated speed at MLSS (S). 

All data processing was performed using custom MATLAB software (version 2021b, Mathworks, Inc., Natick, MA, USA).

A static attitude correction was performed on acceleration data to align the sensor with a global three-dimensional position to enable comparisons between trials and participants [10,44,45].

Initial contact (IC) for each step was identified using methods described in Benson et al. [8]. A step was defined as the duration between consecutive ICs from contralateral feet, and a stride was defined as the duration between consecutive ICs from the ipsilateral foot. Mean and standard deviation of the number of data points in each step were calculated, and those ±2 standard deviations from the mean were labeled as improperly segmented and excluded [8].

Samples were created by selecting five consecutive strides [7,8]. Non-fatigue (NF) and fatigue (FT) conditions were considered the first 5 min of the trial and the final complete 5 min segment (to correspond to RPE sampling), respectively [9]. For example, if a participant terminated the test at 38 min, the period from 30 to 35 min would be defined as the FT condition using a single RPE value (taken at 35 min) to represent the segment. The RPE label for each condition was the value provided at the end of the 5 min segment.

### 2.3. Feature Extraction

Acceleration data were not filtered, and raw signals were maintained for feature creation [9,11,34]. Features were chosen based on previous literature [8,29] and were extracted from the three primary axes (VT, ML, AP) and the resultant (RES) of the acceleration signal: mean, standard deviation, median, 25th percentile, 75th percentile, RMS [5,6,46], maximum, minimum [5,11], and sample entropy (SE) [11,12,47,48,49]. The SE signal stabilizes over 2000 data points [50], and the minimum number of data points in each of our samples was ~3400 (corresponding to a cadence of 180 steps per minute), indicating that there were sufficient data for the calculation. A gait cycle approach, rather than a time sample approach, was used to derive the measurement [12,51]. Additionally, the ratio of single-axis RMS (RMS_VT_, RMS_ML_, RMS_AP_) to RMS_RES_ (RMSR) [34] was extracted. This resulted in 39 features extracted from each sample.

### 2.4. Statistical Analysis

One-way analysis of variance (ANOVA) was performed to compare the following variables across trials: trial time, RPE_NF_, RPE_FT_, change in RPE from NF to FT (ΔRPE), number of strides analyzed for NF, and number of strides analyzed for FT. Additionally, paired t-tests were performed between RPE_NF_ and RPE_FT_ for each trial.

The IMU data were analyzed using two methods: first by comparing MLSS1 and MLSS2 (MM, where speed was the same in both trials) and subsequently comparing all 4 experimental trials (ALL). In both methods, a two-way ANOVA comparing fatigue state and trial (i.e., fatigue state (NF vs. FT) × trial (MLSS1 vs. MLSS2) *or* fatigue state (NF vs. FT) × trial (MLSS1 vs. MLSS2 vs. F vs. S)) was performed for each feature. Additionally, within-fatigue state, between-trial reliability for each feature was evaluated by intraclass correlation coefficients (ICC 2,*k*). Values of <0.5, 0.5–0.75, 0.75–0.9, and >0.9 were interpreted as poor, moderate, good, and excellent, respectively [52]. This was calculated for both MM and ALL methods. 

The level of significance was set at *p* < 0.05 for the statistical tests. Statistical analyses were performed using SPSS version 26 (IBM Corp., Armonk, NY, USA).

## 3. Results

Participants reported a recent training volume of 31.0 ± 21.0 km/week for the previous 3 months and had a recent performance best 10 km time of 44.5 ± 6.3 min. Trial details are presented in Table 1. Except for the F trial, all participants completed at least 30 min of treadmill running during the MLSS1, MLSS2, and S trials (Table 1).

### 3.1. Between-Trial Analysis

There were no significant fatigue state × trial interactions for the MM method. For the ALL method, only 25th percentile (RES) demonstrated a significant fatigue state × trial interaction (*p* < 0.001). There were no main effects for trial for either the MM or ALL methods. The main effects for the fatigue state are summarized in Table 2. Specific values for each feature during each trial and fatigue state are contained in Supplementary Materials Table S1.

### 3.2. Reliability Analysis

**Figure 1:**
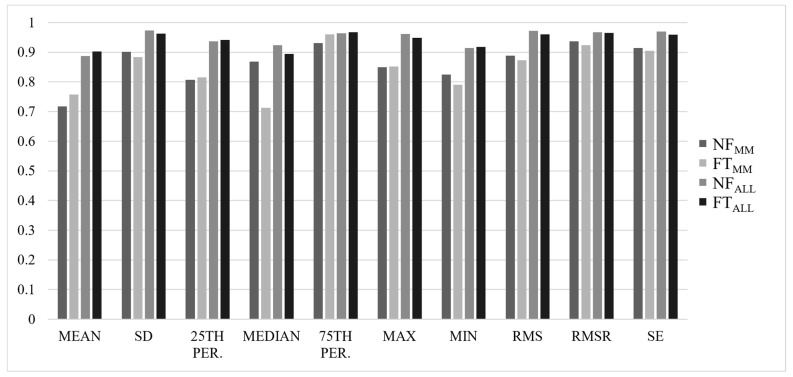
ICC(2,*k*) values for all analyses in the vertical (VT) axis of the acceleration signal. MM = comparison performed between first (MLSS1) and second (MLSS2) trials at MLSS speed, ALL = comparison performed between all four trials; NF = non-fatigued state, FT = fatigued state, SD = standard deviation, RMS = root mean square, RMSR = ratio of root mean square, SE = sample entropy.

**Figure 2:**
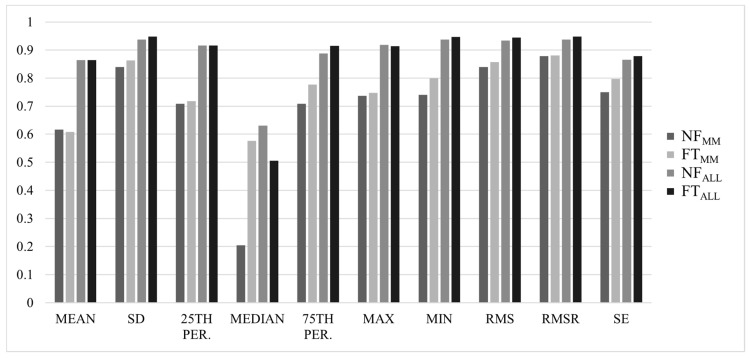
ICC(2,*k*) values for all analyses in the mediolateral (ML) axis of the acceleration signal.

**Figure 3:**
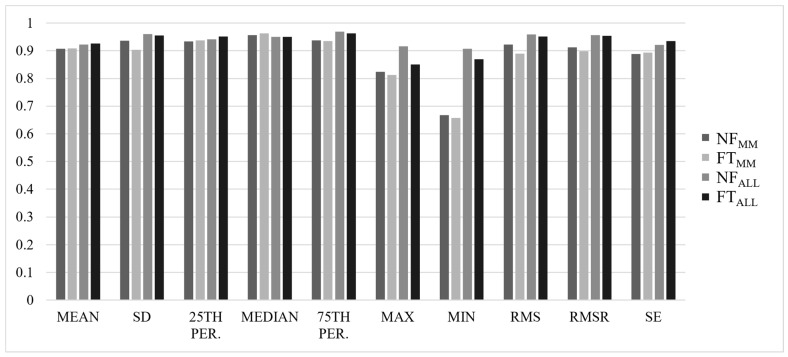
ICC(2,*k*) values for all analyses in the anterior–posterior (AP) axis of the acceleration signal.

**Figure 4:**
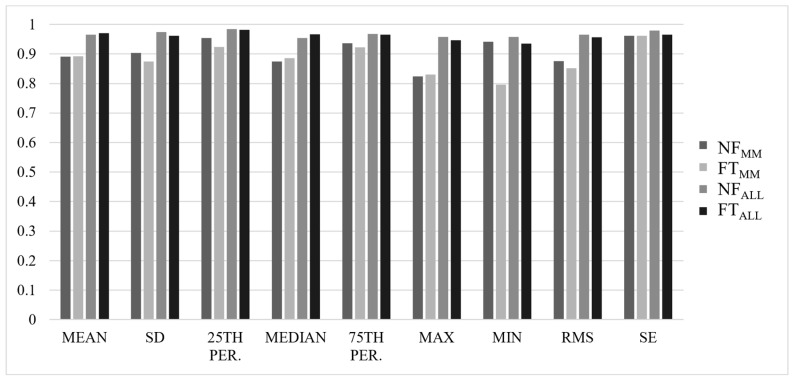
ICC(2,*k*) values for all analyses in the resultant (RES) of the acceleration signal.

Most ICCs were considered good-to-excellent, with ALL values slightly higher than MM values, and NF values slightly higher than FT values (NF_MM_ = 0.839 ± 0.14, FT_MM_ = 0.812 ± 0.24, NF_ALL_ = 0.934 ± 0.06, FT_ALL_ = 0.927 ± 0.08). Results for ICC(2,*k*) values for each axis are summarized in Figure 1, Figure 2, Figure 3 and Figure 4.

## 4. Discussion

The primary finding of the present study was that, in general, the IMU features included in this analysis can be considered highly reliable for both NF and FT conditions. The ICCs reported were mostly good or excellent (ICC > 0.75), and there were no main effects for the trial, indicating that features were similar across days in both NF and FT conditions, even with differing speeds. These results do not support our hypothesis that minor (±5%) variations in speed would reduce the day-to-day reliability of a feature. Although the F trial was significantly shorter than the other trials, there was no main effect for the trial, indicating that the directionality and pattern of fatigue-related alterations are consistent even if the time to onset is different. Therefore, researchers can be reasonably confident that single-trial studies using IMUs to detect fatigue-related changes are representative of the participants’ typical running gait biomechanics irrespective of fatigue state.

Interestingly, for the MM method, 6 of 8 features with ICC values <0.75 were in the ML axis. Although these same features were considered good or excellent when analyzed using the ALL method (except for median), the mean ICC values in the ML axis (NF_ALL_ = 0.88 ± 0.09, FT_ALL_ = 0.88 ± 0.13) were still lower than those in the AP (NF_ALL_ = 0.94 ± 0.03, FT_ALL_ = 0.93 ± 0.04) and VT (NF_ALL_ = 0.95 ± 0.03, FT_ALL_ = 0.94 ± 0.03) axes. The median in the ML axis demonstrated particularly low ICC values (NF_MM_ = 0.21, FT_MM_ = 0.58, NF_ALL_ = 0.63, FT_ALL_ = 0.51); although, there were no significant differences across trials. Previous studies have found the greatest levels of running fatigue-related deviation [28] and sensitivity to disease-related gait changes [53] in the ML axis. Taken with the present results, this may suggest that the musculature controlling ML movements is the most susceptible to fatigue, and this increased variability may contribute to lower reliability.

The results from the NF state are consistent with previous findings on the reliability of the acceleration RMS signal, which have reported ICC values of 0.89–0.98, 0.95–0.97, 0.97, and 0.90–0.99 for the RMS_VT_, RMS_ML_, RMS_AP_, and RMS_RES_, respectively [32,34]. These values align with our findings, where ICC values using the ALL method were 0.97, 0.93, 0.96, and 0.97 for RMS_VT_, RMS_ML_, RMS_AP_, and RMS_RES_, respectively. However, the values from the previous studies and the ALL method were somewhat higher than those of the MM method (RMS_VT_ = 0.89, RMS_ML_ = 0.84, RMS_AP_ = 0.92, RMS_RES_ = 0.88). To our knowledge, no other accelerometer-derived statistical features have been previously analyzed for reliability, so it is not possible to compare other features to previous results.

Most features demonstrated a significant main effect for fatigue state, confirming that these measures are largely sensitive to fatigue-related changes in biomechanical alterations during running, and that the high reliability during FT was not only due to a lack of changes from NF to FT. The observed changes in biomechanics with fatigue were also generally consistent with previous literature. For example, the acceleration RMS values in the current study were observed to increase in all axes, which is consistent with Schütte et al. [12], and somewhat consistent with Le Bris et al. [28], who reported increases in RMS_ML_ only. A change in the RMSR was observed in all axes. RMSR_VT_ decreased, while RMSR_ML_ and RMSR_AP_ values increased; although, for RMSR_AP_, only the ALL method was significant. These results are similar to previous results [47,54], such as Winter et al. [55], who reported decreased RMSR_VT_ and increased RMSR_ML_. Schütte et al. [48] also reported an increase in RMSR_ML_, and in another study, [12] reported increased RMSR_ML_ and RMSR_AP_. These slightly different results may be due to differences in protocol and statistical methodology. For example, while Winter et al. [55] used an all-out 8 km run, the others used exhaustive protocols that lasted only 4–20 min [12,28,48]. These shorter protocols involved running to exhaustion, indicating subjects were likely running well above MLSS (i.e., unsustainable intensity), which could have produced more variability in their responses [38]. The relatively consistent observation of increased RMS_ML_ and RMSR_ML_ supports the earlier suggestion that the reliability of ML features may be lower due to increases in variability with fatigue.

There were also main effects for fatigue state for SE, with SE_VT_ and SE_RES_ increasing significantly, SE_ML_ decreasing significantly, and no changes observed in SE_AP_. These results are similar to Pla et al. [47], who demonstrated increases in SE_VT_ with fatigue, but also observed an increase in SE_AP_. However, Schütte et al. [12] reported contrary results, with no fatigue-related changes in SE_VT_ or SE_ML_, but significant increases in SE_AP_. Interestingly, in another study, these same authors [48] reported fatigue-related decreases in SE_ML_, but only for those with a history of medial tibial stress syndrome. Higher SE indicates lower predictability in the signal, which may represent neuromuscular re-organization [51]. An increase in SE with running fatigue has been interpreted as a protective neuromuscular mechanism to avoid pain [12], whereas lower SE has been associated with unhealthy and frail states [56]. Whether the present decrease in SE_ML_ portends specific injury risk or reflects injury history is beyond the scope of the study but should continue to be investigated.

Specific changes in RMS, RMSR, and SE are discussed here given their previous use in the literature, but caution is warranted in over-interpreting any single significant feature here. Between the MM and ALL methods, 78 features were compared; given that α = 0.05, ~4 effects would be significant due to chance [57]; although, the general pattern of significant differences between fatigue states indicates that biomechanical adjustments were made over the course of the trial. However, single feature interpretation is not necessarily required, as most of these features are used as inputs for machine learning algorithms in practice [29,30,31]. Therefore, the reliability of the features is more important than the individual features’ sensitivity to fatigue.

Additional limitations are acknowledged. First, no differences were found between trials when speeds were different, which contradicts previous findings from studies investigating non-fatigued biomechanics [33,34]. The absence of speed-related differences is likely due to smaller perturbations in speed than previously reported. While this protocol only altered speed ±5%, others used ranges of 8–16 km/h (2.22–4.44 m/s) [34], or ~±15% changes [33]. Future studies should investigate the effect of larger changes in speed on fatigue-related biomechanical adjustments. Second, Benson et al. [58] and Ahamed et al. [59] previously reported that a consistent running pattern can be determined using IMU data from 4 to 5 runs, whereas the current study only compared between-day reliability. Third, the study design was highly controlled, and data were collected in a laboratory setting. Although the treadmill was useful for maintaining speed, multiple studies have demonstrated differences in treadmill and overground running biomechanics [60,61], as well as differences in fatigue-related changes [9,57] so these results may not be generalizable to outdoor running. Fourth, the features investigated here were not comprehensive. Future studies should investigate the fatigue-related reliability of discrete features (e.g., ground contact time, step length) and features derived from the other instruments in the IMU (e.g., gyroscope). Fifth, the RPE at trial termination (14.1–16.8) was lower than others have previously defined as “fatigue” (i.e., 17) [62,63,64]. However, our RPE_FT_ values were consistent with other studies reporting running fatigue [25,29] and represented a significant increase from the RPE_NF_ in each trial. Sixth, possible signal drift is an important limitation of IMUs. However, the current study only utilized the accelerometer readings, which are less affected by drift than the other sensors, and the temperature was kept relatively constant throughout each trial [65]. Finally, some trials were terminated due to volitional exhaustion, while others were terminated due to the stipulated maximum duration. These two “types” of trials may have generated different fatigue-related adjustments; although, this likely would have shown up as a main effect of the trial, as only one participant was able to complete 45 min for all trials.

In summary, to our knowledge, this is the first study to present evidence that fatigue-related alterations to biomechanics are consistent day-to-day. The results of the current study suggest that the commonly used features extracted from a CoM-mounted IMU during treadmill running are reliable in both NF and FT conditions, are not sensitive to slight (±5%) alterations in speed, and are generally sensitive to changes in fatigue state; although, features extracted from the ML axis should be interpreted with greater caution. Further study is needed to determine if these results are generalizable to overground running, or if larger changes in speed have different effects.

## Figures and Tables

**Table 1:** Values for trial variables. MLSS1 = first trial at speed at MLSS, MLSS2 = second trial at speed at MLSS, F = trial at 5% faster than speed at MLSS, S = trial at 5% slower than speed at MLSS, NF = non-fatigued state, FT = fatigued state.

	MLSS1	MLSS2	F	S
Treadmill speed (m/s)	3.35 ± 0.4	3.35 ± 0.4	3.53 ± 0.4	3.17 ± 0.4
Number of strides used (NF)	376.8 ± 119.2	390.5 ± 69.5	411.5 ± 127	395.5 ± 109.5
Number of strides used (FT)	375.5 ± 71.5	387.5 ± 53.5	366 ± 77	362.5 ± 61
Trial time (min) *	39.8 ± 6.6 ^@^	37.7 ± 7.3 ^@^	28.8 ± 6.9	42.0 ± 5.6 ^@^
RPE_NF_	11.5 ± 1.7	11.4 ± 1.8	12.5 ± 1.5	11.0 ± 1.5
RPE_FT_ *	15.6 ± 1.6 ^#,^^	15.3 ± 1.6 ^#,^^	16.8 ± 1.3 ^†,‡,#,^^	14.1 ± 1.7 ^^^
ΔRPE	4.1 ± 2.0	3.9 ± 2.0	4.3 ± 1.7	3.1 ± 1.6

* Significant main effect (*p* < 0.05); ^@^ significantly greater than F trial (*p* < 0.05); ^†^ significantly greater than MLSS1 trial (*p* < 0.05); ^‡^ significantly greater than MLSS2 trial (*p* < 0.05); ^#^ significantly greater than S trial (*p* < 0.05), ^^^ significantly greater than NF state (*p* < 0.05).

**Table 2:** Main effects for fatigue state in each axis of the acceleration signal. Direction of change indicates differences from NF to FT. Significant *p*-values in bold. VT = vertical, ML = mediolateral, AP = anterior–posterior, RES = resultant; MM = comparison performed between MLSS1 and MLSS2, ALL = comparison performed between all four trials. RMS = root mean square, RMSR = ratio of root mean square, SE = sample entropy.

Axis	Feature	MM		ALL	
		*p* Value	Direction of Change	*p* Value	Direction of Change
VT	Mean	**0.022**	-	**0.009**	-
	St. deviation	**0.032**	+	**0.006**	+
	25th percentile	0.091		**0.003**	-
	Median	**0.014**	+	**0.001**	+
	75th percentile	0.741		0.479	
	Max	**0.003**	+	**0.002**	+
	Min	0.281		**0.037**	-
	RMS	0.075		**0.023**	+
	RMSR	**<0.001**	-	**<0.001**	-
	SE	**0.001**	+	**0.001**	+
ML	Mean	0.098		0.115	
	St. deviation	**<0.001**	+	**<0.001**	+
	25th percentile	0.152		**0.025**	-
	Median	0.207		0.123	
	75th percentile	0.492		0.212	
	Max	**<0.001**	+	**<0.001**	+
	Min	**0.004**	-	**<0.001**	-
	RMS	**<0.001**	+	**<0.001**	+
	RMSR	**<0.001**	+	**<0.001**	+
	SE	**<0.001**	-	**<0.001**	-
AP	Mean	**0.001**	-	**<0.001**	-
	St. deviation	**0.007**	+	**<0.001**	+
	25th percentile	**0.002**	-	**<0.001**	-
	Median	0.699		0.241	
	75th percentile	0.157		**0.027**	+
	Max	0.123		0.514	
	Min	**0.009**	-	**<0.001**	-
	RMS	**0.008**	+	**0.001**	+
	RMSR	0.078		**0.028**	+
	SE	0.136		0.07	
RES	Mean	**<0.001**	+	**<0.001**	+
	St. deviation	**0.016**	+	**0.009**	+
	25th percentile	**0.001**	+	0.06	+
	Median	0.169		**0.005**	+
	75th percentile	0.38		0.346	
	Max	**0.003**	+	**0.004**	+
	Min	0.136		0.601	
	RMS	**0.001**	+	**<0.001**	+
	SE	**0.017**	+	**0.01**	+

## Data Availability

The data generated and/or analyses for to the current study are available from the corresponding author upon reasonable request.

## Supplementary Materials

The following supporting information can be downloaded at: https://www.mdpi.com/article/10.3390/s22114129/s1, Table S1: Mean values used for reliability and between-trial analysis.
