# Supplementary material for: Between-Day Reliability of Commonly Used IMU Features during a Fatiguing Run and the Effect of Speed
Source: Sensors (Basel). 2022 May 29;22(11):4129. doi: 10.3390/s22114129 (PMC9185649; doi:10.3390/s22114129)
Supplement: Supplementary file 1 [file sensors-22-04129-s001.zip › sensors-1718321-supplementary.pdf]

		MLSS 1		MLSS 2		F		S	
Axis	Feature	NF	FT	NF	FT	NF	FT	NF	FT
VT	mean	9.53 ± 0.27	9.51 ± 0.29	9.53 ± 0.37	9.50 ± 0.41	9.56 ± 0.24	9.55 ± 0.26	9.57 ± 0.25	9.55 ± 0.26
	SD	11.73 ± 2.19	11.94 ± 2.27	11.91 ± 2.50	12.10 ± 2.41	11.94 ± 2.02	12.05 ± 1.89	11.66 ± 1.96	11.87 ± 1.89
	25th percentile	-0.55 ± 1.16	-0.61 ± 1.08	-0.37 ± 1.23	-0.54 ± 1.32	-0.56 ± 1.12	-0.71 ± 1.04	-0.43 ± 0.80	-0.65 ± 0.91
	median	7.31 ± 1.49	7.66 ± 1.28	7.23 ± 1.42	7.52 ± 1.24	7.10 ± 1.66	7.42 ± 1.55	7.50 ± 1.50	7.56 ± 1.44
	75th percentile	18.20 ± 1.82	18.29 ± 1.69	18.41 ± 2.05	18.04 ± 2.11	18.62 ± 1.83	18.38 ± 1.93	18.73 ± 1.64	18.33 ± 1.72
	max	57.38 ± 28.23	62.03 ± 29.64	60.89 ± 29.92	65.14 ± 31.75	56.36 ± 22.91	58.90 ± 21.76	56.45 ± 26.94	59.12 ± 25.15
	min	-7.20 ± 2.59	-7.47 ± 2.48	-7.91 ± 2.99	-7.94 ± 2.77	-7.75 ± 1.68	-7.82 ± 1.82	-7.36 ± 2.59	-7.76 ± 2.73
	RMS	15.17 ± 1.79	15.32 ± 1.88	15.31 ± 2.12	15.44 ± 2.07	15.34 ± 1.66	15.41 ± 1.56	15.13 ± 1.63	15.27 ± 1.58
	RMSR	0.87 ± 0.05	0.86 ± 0.06	0.86 ± 0.068	0.85 ± 0.07	0.86 ± 0.05	0.86 ± 0.05	0.88 ± 0.06	0.87 ± 0.06
	SE	0.0059 ± 0.0020	0.0064 ± 0.0023	0.0063 ± 0.0024	0.0066 ± 0.0025	0.0058 ± 0.0019	0.0060 ± 0.0020	0.0056 ± 0.0020	0.0058 ± 0.0022
ML	mean	0.71 ± 0.74	0.68 ± 0.75	0.48 ± .78	0.47 ± 1.19	0.40 ± 0.74	0.39 ± 0.74	0.48 ± 1.03	0.47 ± 0.71
	SD	5.40 ± 1.33	5.83 ± 1.44	6.03± 1.82	6.45 ± 1.92	5.93 ± 1.29	6.17 ± 1.29	5.28 ± 1.25	5.62 ± 1.30
	25th percentile	-2.17 ± 0.79	-2.26 ± 0.84	-2.49 ± 1.30	-2.56 ± 1.15	-2.53 ± 1.13	-2.62 ± 1.09	-2.17 ± 0.96	-2.25 ± 1.01
	median	0.56 ± 0.52	0.51 ± 0.45	0.38 ± 0.52	0.33 ± 0.62	0.36 ± 0.45	0.34 ± 0.52	0.40 ± 0.53	0.36 ± 0.48
	75th percentile	3.38 ± 1.28	3.40 ± 1.08	3.32 ± 1.69	3.38 ± 1.58	3.22 ± 0.95	3.23 ± 1.01	3.02 ± 1.31	3.10 ± 1.29
	max	24.11 ± 11.29	26.48 ± 12.82	25.32 ± 11.24	27.59 ± 10.88	23.51 ± 7.96	24.69 ± 7.99	22.25 ± 9.62	24.20 ± 9.77
	min	-17.37 ± 7.15	-19.96 ± 8.82	-23.33 ± 14.68	-23.88 ± 13.51	-21.71 ± 9.71	-22.56 ± 9.90	-18.68 ± 10.40	-20.08 ± 10.12
	RMS	5.49 ± 1.35	5.91 ± 1.44	6.14 ± 1.87	6.55 ± 1.97	5.99 ± 1.28	6.23 ± 1.28	5.39 ± 1.26	5.72 ± 1.30
	RMSR	0.31 ± 0.06	0.33 ± 0.06	0.34 ± 0.08	0.36 ± 0.08	0.33 ± 0.06	0.35 ± 0.06	0.31 ± 0.06	0.33 ± 0.06
	SE	0.026 ± 0.009	0.023 ± 0.008	0.023 ± 0.009	0.020 ± 0.008	0.023 ± 0.007	0.022 ± 0.008	0.026 ± 0.008	0.023 ± 0.007

AP	mean	0.66 ± 0.78	0.55 ± 1.80	0.32 ± 1.76	0.21 ± 1.80	0.65 ± 1.71	0.54 ± 1.74	0.37 ± 1.64	0.28 ± 1.68
	SD	5.89 ± 1.70	6.20 ± 1.95	6.10 ± 1.78	6.26 ± 1.83	6.21 ± 1.68	6.34 ± 1.62	5.55 ± 1.74	5.74 ± 1.79
	25th percentile	-3.50 ± 3.46	-3.79 ± 3.63	-4.10 ± 3.50	-4.26 ± 3.59	-3.95 ± 3.33	-4.07 ± 3.26	-3.57 ± 3.41	-3.82 ± 3.27
	median	0.20 ± 1.78	0.22 ± 1.72	-0.04 ± 1.83	-0.09 ± 1.74	0.20 ± 1.60	0.082 ± 1.69	0.09 ± 1.51	0.092 ± 1.57
	75th percentile	4.08 ± 0.80	4.16 ± 0.90	4.06 ± 0.87	4.13 ± 0.83	4.36 ± 0.79	4.35 ± 0.77	3.78 ± 0.81	3.93 ± 0.96
	max	23.84 ± 8.60	25.22 ± 10.89	21.95 ± 8.36	22.75 ± 8.35	24.07 ± 8.39	23.35 ± 7.96	20.40 ± 7.01	20.07 ± 6.92
	min	-22.30 ± 17.02	-25.13 ± 19.51	-23.16 ± 13.88	-25.47 ± 13.41	-19.74 ± 13.02	-20.99 ± 11.99	-19.98 ± 14.83	-22.12 ± 14.85
	RMS	6.19 ± 1.59	6.48 ± 1.89	6.35 ± 1.75	6.50 ± 1.85	6.48 ± 1.57	6.59 ± 1.56	5.78 ± 1.78	5.96 ± 1.85
	RMSR	0.35 ± 0.08	0.36 ± 0.09	0.36 ± 0.09	0.36 ± 0.10	0.36 ± 0.08	0.37 ± 0.08	0.33 ± 0.09	0.34 ± 0.10
	SE	0.025 ± 0.010	0.026 ± 0.012	0.026 ± 0.011	0.027 ± 0.013	0.024 ± 0.010	0.025 ± 0.010	0.028 ± 0.013	0.028 ± 0.014
RES	mean	13.90 ± 1.17	14.16 ± 1.15	14.19 ± 1.32	14.42 ± 1.27	14.32 ± 1.10	14.49 ± 1.07	13.70 ± 0.98	13.97 ± 1.03
	SD	10.34 ± 2.12	10.66 ± 2.33	10.70 ± 2.46	10.92 ± 2.40	10.49 ± 1.96	10.57 ± 1.81	10.31 ± 1.95	10.46 ± 1.84
	25th percentile	5.65 ± 1.57	5.90 ± 1.48	5.89 ± 1.78	6.17 ± 1.67	5.97 ± 1.63	6.17 ± 1.54	5.97 ± 1.63	5.93 ± 1.61
	median	10.89 ± 1.49	10.99 ± 1.28	10.81 ± 1.61	10.98 ± 1.62	11.18 ± 1.51	11.43 ± 1.41	10.56 ± 1.39	10.72 ± 1.52
	75th percentile	20.76 ± 1.30	20.70 ± 1.29	20.96 ± 1.45	20.85 ± 1.30	21.10 ± 1.25	20.97 ± 1.46	20.70 ± 1.35	20.75 ± 1.28
	max	63.89 ± 31.78	68.50 ± 33.48	66.34 ± 30.98	70.83 ± 31.72	61.51 ± 24.44	63.23 ± 22.29	60.90 ± 28.92	63.95 ± 26.92
	min	0.83 ± 0.57	0.73 ± 0.39	0.97 ± 0.78	0.91 ± 0.65	0.84 ± 0.45	0.89 ± 0.53	0.93 ± 0.78	0.96 ± 0.73
	RMS	17.38 ± 1.98	17.78 ± 2.13	17.84 ± 2.29	18.16 ± 2.16	17.80 ± 1.77	17.98 ± 1.65	17.19 ± 1.76	17.49 ± 1.65
	SE	0.0084 ± 0.0032	0.0088 ± 0.0031	0.0086 ± 0.0033	0.0091 ± 0.0031	0.0086 ± 0.0032	0.0088 ± 0.0030	0.0080 ± 0.0027	0.0083 ± 0.0033

Table S1. Mean values used for reliability and between-trial analysis. MLSS1 = first trial at MLSS speed, MLSS2 = second trial at MLSS speed, F = fast (MLSS speed +5%) trial, S = slow (MLSS speed -5%) trial, VT = vertical, ML = mediolateral, AP = anterior-posterior, RES = resultant; NF = non-fatigued state, FT = fatigued state, SD = standard deviation, RMS = root mean square, RMSR = ratio of root mean square, SE = sample entropy
